# Editorial: Living labs and open innovation approaches to scale impact for human wellbeing

**DOI:** 10.3389/fpubh.2024.1378932

**Published:** 2024-03-22

**Authors:** Ann Borda, Dimitri Schuurman, Sonja Pedell, Francesca Spagnoli, Evdokimos Konstantinidis

**Affiliations:** ^1^Centre for Health Policy, The University of Melbourne, Parkville, VIC, Australia; ^2^IMEC, Leuven, Belgium; ^3^Swinburne Living Lab, Swinburne University of Technology, Hawthorn, VIC, Australia; ^4^European Network of Living Labs, Brussels, Belgium; ^5^Medical Physics & Digital Innovation Lab, Aristotle University of Thessaloniki, Thessaloniki, Greece

**Keywords:** living labs approach, quadruple helix, wellbeing, quintuple helix, innovation lifecycle, emerging technologies, ecosystems, co-design

This Research Topic of Frontiers in Public Health focuses on different innovation aspects related to Living Labs in various thematic contexts, collectively addressing ways of scaling impact for human wellbeing. Living Labs are powerful instruments supporting healthy communities, cities and regions in their transition toward sustainable and resilient futures with the facilitation of open and inclusive innovation (1–4). As orchestrators of open innovation environments, Living Labs aim to involve all relevant stakeholders to co-create concrete, long-term solutions based on real-life problems with the goal to scale-up eventually (5, 6). The Living Lab innovation model as an emerging practice centering on open innovation has particular resonance in contexts that have wellbeing and quality of life at their heart with a focus on the role of human-centered technologies supporting this goal (7, 8). The articles in this Research Topic are best practice examples in capturing the breadth and complexity that is necessary to achieve new co-created solutions as represented by the 11 articles contributed by 72 authors.

Living lab networks, like the European Network of Living Labs (ENoLL), have a global presence and provide a collaborative approach in bringing together stakeholders to explore and design socio-technological solutions addressing real-world challenges. A critical difference to other forms of innovation or technology incubation is that the living lab approach centers the design and evaluation of these innovations directly with users (e.g., citizens, clients, patients) so they can shape the innovation to their actual life and work environments based on needs, lived experiences and expectations (9, 10).

The aim of this Research Topic is to raise the awareness and opportunities of current international research and practice in the intersection of Living Lab models and digital public health and human wellbeing across communities, cities and regions. The collection of papers in this Research Topic encompasses original research contributions, as well as selected and reworked papers from the Open Living Lab Days' 2022 top research session, ENoLL's yearly innovation conference. Together, they demonstrate a range of diverse and accessible perspectives, including stakeholder engagement in Living Labs, scaling of healthcare solutions, infrastructure in Living Labs, and Living Labs in the light of a broader societal context.

The first article in the collection authored by Fotis et al., entitled “*Co-creation in a digital health living lab: A case study*,” is a single use case description of stakeholder engagement to co-develop strategies in self-managed care for older adults. Specifically the community based Digital Health Living Lab (DHLL) is a partnership between the University of Brighton and the Brighton and Hove City Council supporting an open innovation process with multiple stakeholders, such as older adults, contributing to the co-development of a digital health solution led by a small business enterprise. Lessons learned provide some insights into the co-benefits of testing in a real-life environment, cost benefits of setting up a living lab within the community, and similar advantages for SMEs utilizing a DHLL to engage end users directly with their solutions.

The second article is entitled: “*“Loved ones are not “visitors” in a patient's life” - the importance of including loved ones in the patient's hospital stay: an international Twitter study of #HospitalsTalkToLovedOnes in times of COVID-19,”* and is written by Hribersek et al.. This article differs from the ‘traditional' Living Lab approach, presenting an interesting outlook on the role of family and friends in the life of hospital patients. This article studied 8 months of Twitter interactions using a variety of techniques, including thematic analysis, term frequency and Markov chain analysis. The study looked at 4,412 unique tweets and interactions by 7,040 Twitter users originating from 142 countries. Results indicated the important role for communication between patients, patients' loved ones and hospitals. The study concluded that support is needed during a patient's hospital journey, irrespective of the pandemic context caused by COVID-19. Patient empowerment and transparent communication improve the hospital experience and patient safety. Moreover, the outcomes from the study underline the need for family-centered care in the context of adult nursing clinical practice.

The third article is titled “*Innovation through the Quintuple Helix in living labs: lessons learned for a transformation from lab to ecosystem,”* from Merino-Barbancho et al.. In the digital age, prioritizing citizen-centric innovation is imperative for cultivating resilient and collaborative communities. Living Labs, and notably their use of the Quintuple Helix model, have emerged as an effective strategy for user-centered design and co-creative innovation. This study highlights the successful integration of the Quintuple Helix in the revitalization of LifeSpace, managed by the Polytechnic University of Madrid, drawing insights from the ACTIVAGE pilot. Tested at the Madrid Deployment Site with over 350 participants, the model fosters a sense of community known as MAHA. The Living Lab infrastructure combined with the Quintuple Helix model has been proven successfully by incorporating three environments: THE LAB for planning, THE CLUB for validating solutions and THE NEIGHBORHOOD for real-life implementation. This research underscores the Quintuple Helix's role in facilitating coordinated participation from diverse stakeholders, transcending traditional boundaries in research and innovation processes.

The fourth article “*“A living lab within a lab”: approaches and challenges for scaling digital public health in resource-constrained settings”* by Mukherjee et al. address the process of establishing Living Labs and their innovation processes beyond Europe, and in particular in low- and middle-income countries within the context of healthcare. This article investigates the challenges linked to building appropriate digital solutions for local health challenges and scaling them to other public health facilities through ongoing empirical work in India and identifies three key domains of analysis: (1) the process of establishing an enabling structure of a “living lab within a lab”; (2) leveraging the capabilities offered by free and open-source digital technologies; and (3) the driving impetus to scaling through agile and co-constructed technical support. The study findings acknowledge that processes need to be adapted to context-based and resource-constrained public health systems and that resource proximity has a further enabling role to achieve an effective “lab within a lab” model. However, any future studies should ideally examine how a model can be made more robust and sustainable.

The fifth article is titled: “*Living labs for civic technologies: a case study. Community infrastructuring for a volunteer firefighting service*,” from Viano et al.. This research delves into the increasing use of digital technologies within Living Labs, specifically examining their role in facilitating co-production processes for wellbeing-related public services. The study focuses on a case from the European project NLAB4CIT, situated in Kaisariani, Greece. Emphasizing community engagement, the report applies participatory design methods within an “infrastructuring” framework, reimagining the Living Lab model as community infrastructure and digital tools as civic technologies. It explores the initial co-design phases, offering insights into socio-technical challenges encountered. Strengths identified include an active community, a sustained collaboration space between researchers and citizens, and a civic approach to technology. Challenges outlined encompass the role of public administration, the degree of co-design and co-development of technologies, and issues such as internet accessibility. The overarching aim of this research is to furnish a valuable overview for other Living Labs involved in digital co-production.

The sixth article is entitled “*Perceived factors informing the pre-acceptability of digital health innovation by aging respiratory patients: a case study from the Republic of Ireland,”* by Byrne et al.. The goal of this study is to inform future decision-making among respiratory patients by identifying relevant themes to respiratory care and digital health experts in the Republic of Ireland. The end goal is to facilitate engagement with and appropriate use of digital health innovation (DHI). To this end, semi-structured interviews were conducted which revealed that privacy, trustworthiness, utility, equality and data literacy are key themes to take into account. A Living Lab approach can support creating effective DHI's for respiratory care, guided by multi-stakeholder involvement and by the Quintuple Helix Hub framework. In conclusion to this study, the authors advocate for more research to bridge the gap between bottom-up end-user engagement on the one hand and top-down digital health policies on the other so that an effective and safe use of DHI is facilitated.

The seventh article is entitled “*A co-design living labs philosophy of practice for end-to-end research design to translation with people with lived-experience of mental ill-health and carer/family and kinship groups*,” by Palmer et al. (on behalf of the Co-Design Living Labs Program Members, The University of Melbourne). This article promotes the development of a suitable infrastructure in the health sector and focuses on the lived-experience of people when translating research into practice in the area of mental ill-health. The article steps the reader through the evolution of the Co-design Living Labs program, a community-based embedded approach with 2,000 members. The authors emphasize a philosophy of practice for working with people with lived-experience called “togetherness by design.” The retrospective demonstrates how an initially researcher-driven model can share decision power to create change and have people with lived experiences move into co-researcher roles. Eight mechanisms constitute a theoretical model to frame research co-design activities and to provide space for continuous learning in the Living Lab.

The eighth article is entitled “*How to bridge the nurse innovation–diffusion gap? An in-depth case study of Create4Care*,” by Rigtering et al. (Utrecht University) and aims to scale innovative solutions for nurses. This research applies a qualitative approach studying a medical makerspace at the largest academic hospital in the Netherlands to reduce diffusion shortage. Results indicate that innovations are prevented from broadening and being developed further due to a range of personal, organizational, regulatory, and market barriers. The authors suggest that the development of innovation ecosystems can take on the role of progressing the innovation and diffusion process. Within this ecosystem perspective the main two beneficial elements are (i) support systems that can lead the development and diffusion of innovations and (ii) actors who integrate their functional specializations. The research contributes to theory and practice of making innovations available for the broader medical practice.

The ninth article has the title “*Social system design methodology for transitioning to a new social structure: holistic urban living lab approach to well-being and a sustainable city,”* authored by Kimura et al. focuses on the policy work and community interventions by the urban living lab - Center for Person-Centered Ningen, Omuta (PONI PONI) based in Omuta City, Fukuoka Prefecture in Japan. PONI PONI was established in collaboration with the public and private sectors as an “organization that is both independent and embedded” in the existing social system, crossing vertical sectors and domains to seek effective integration of two different policy areas; namely community-based comprehensive care and regional development. Central to the research is an examination of a social system design methodology used by the living lab to propose a novel way of perceiving social systems and practitioner attitudes, and supporting a process model of social system design. To test the validity and agility of the methodology, two case studies are analyzed involving long-term care prevention and employment practices related to persons with disabilities. The application of the methodology amplifies that existing social systems are prone to fundamental problems due to their cyclical structure and vertical divisions. To overcome this, the use of policy background analysis to clarify existing concepts can result in a refreshed view of social system concepts. Subsequently the support of bottom-up practices to operationalize these concepts can begin to effectively transform social systems.

The tenth article “*Grand challenges and living labs: toward achieving the sustainable development goals,”* an opinion piece by Molnar et al. brings together perspectives from a multidisciplinary multinational author team (Swinburne University of Technology, Karlshochschule International University and University College London) on the opportunities of the Living Lab approach for realizing substantial and sustainable change. Living Labs are seen as suitable instruments to achieve the Sustainable Development Goals (SDGs) due to their ability to support holistic solutions, encourage a continuum of learning and development and incorporate participatory design for stakeholders and ‘everyone' to achieve transformation in the world. Specifically in regard to the complexity of sustainable innovative solutions, Living Labs are a bridge between global ambition and local necessity and its social impact process of partnership (through coordination, collaboration and co-creation). As such Living Labs can directly contribute to an innovation lifecycle of piloting, implementation, and evaluation that can be scaled more quickly and aligned with the SDG required reporting and monitoring mechanisms (e.g., place-based data collection).

The final article is titled: “*Urban living labs as innovation infrastructure for local urban intervention acceleration and student social learning: the impacts on community wellbeing in Heerlen,”* from Blezer et al.. Cities increasingly use urban experiments to address societal challenges and integrate urban planning with citizen needs. This study focuses on the impacts of placemaking and Urban Living Labs (ULLs) on creating healthy environments and fostering transdisciplinary learning. The Aurora transformation process in Heerlen-North's GMS neighborhood serves as a case study for socio-urban challenges in one of 16 Dutch neighborhoods. The research highlights two key outcomes of ULLs as crucial infrastructure for fostering innovation and community wellbeing. ULLs offer an alternative spatial planning approach for areas with severe social-urban conditions, addressing public health equity and socio-economic determinants. Additionally, ULLs serve as educational innovation infrastructure, addressing societal issues like loneliness and social exclusion. The article emphasizes its novelty, discusses findings, and outlines implications for theory, practice, policy, and research, advocating for citizen-centric, experiment-driven approaches in urban development for healthier, more resilient communities.

Reflecting on the Research Topic, the published research which comprises this collection addresses a significant gap in our understanding of the extent to which Living Lab approaches in the design and development of solutions can solve complex problems in our society and scale them within large ecosystems, particularly in sustainable ways through emerging technologies. For instance, both diverse and connected ecosystems are represented in the papers, such as public health, aged care, smart cities, rural areas, transportation and social structures, all of which are variously supported through open innovation, sustainability, and socio-technical frameworks. A key pillar of the Living Labs model, demonstrated in the collective research, is the richness of collaborative methods of participatory and experience-based co-design, co-creation, evaluation, Quintuple Helix, and social system design, among other multi-stakeholder processes across the innovation lifecycle. Critically, the Research Topic highlights that such characteristics of Living Labs are integral to real-world problem solving and validated through exemplars of positive and measurable impacts on the health of communities, societal and individual wellbeing.

## Author contributions

AB: Writing – review & editing, Writing – original draft. DS: Writing – review & editing, Writing – original draft. SP: Writing – review & editing, Writing – original draft. FS: Writing – review & editing, Writing – original draft. EK: Writing – review & editing, Writing – original draft.
